# The evaluation of the mitochondrial genomics in Atlantic salmon (*Salmo salar*)

**DOI:** 10.1080/23802359.2018.1424575

**Published:** 2018-03-09

**Authors:** Mohammad Aghajanpour Ghasemabad, Abolhasan Rezaei

**Affiliations:** The Department of Genetics, Faculty of Biological Sciences, Islamic Azad University, Tonekabon Branch, Iran

**Keywords:** *Salmo salar*, mitochondrial genomic, evolutionary analysis

## Abstract

The mitochondrial genomic of Atlantic salmon (*Salmo salar*) was sequenced and deposited in GenBank accession no. LC012541.1. In this study for the evolutionary analysis of Mtgenomic between *S. salar* and other salmonid species such as *S. t. fario*, *S.t. trutta and S.t. caspius* were conducted by using MEGA Software Version 7. The results showed that there is a major homology between *S. salar* and other salmonids like *S.t. fario* and *S.t. trutta*, and we can see it in Figure 1 showing the close relationship between them.

Salmonidae species only have one family in the name of salmonids including 70 species. The Atlantic salmon (*Salmo salar*) is a species of the ray-finned fish in the family Salmonidae. It is found in the northern Atlantic Ocean and rivers flowing into the north Atlantic in the North Pacific Ocean. Several studies have been performed to find out the relationship between phenotypic traits of salmonid species (*Salmo trutta lacustris* and *Salvenilus alpines* (Pakkasmaa and Piiorenen 2001) *Salmo trutta* (Oadri 1959; Blanc et al. 1982)

*Salmo trutta caspius* (Rezaei et al. 2017) salmon species, which were collected from winter to summer in 2015, were three years old.

The total genomic DNA extracted from tissue taken from fresh specimens that was stored in −20 °C in the refrigerator from Marine Biology of Laboratory for Ecology and Systematic in Islamic Azad University Tonekabon Branch, Tonekabon-Iran. The DNA isolation was carried out using the phenol–chloroform protocol (Sambrook and Russel 2001). After conducting the PCR technique, the universal primers were designed; thereby the PCR product was carried out for sequencing. Mtgenomic in *S. salar* was deposited in GenBank accession no. LC012541.1.

The evolutionary analyses were conducted by using MEGA Software Version 7 (Tamura and Nei 1993). The analysis involved 50 nucleotide sequences. There were 16,622 positions in the final dataset. The results showed that there is a major homology between *S. salar* and other salmonids like *S.t. fario* and *S.t. trutta*. They are shown in Figure 1, and it shows the close relationship between them.

**Figure 1.**
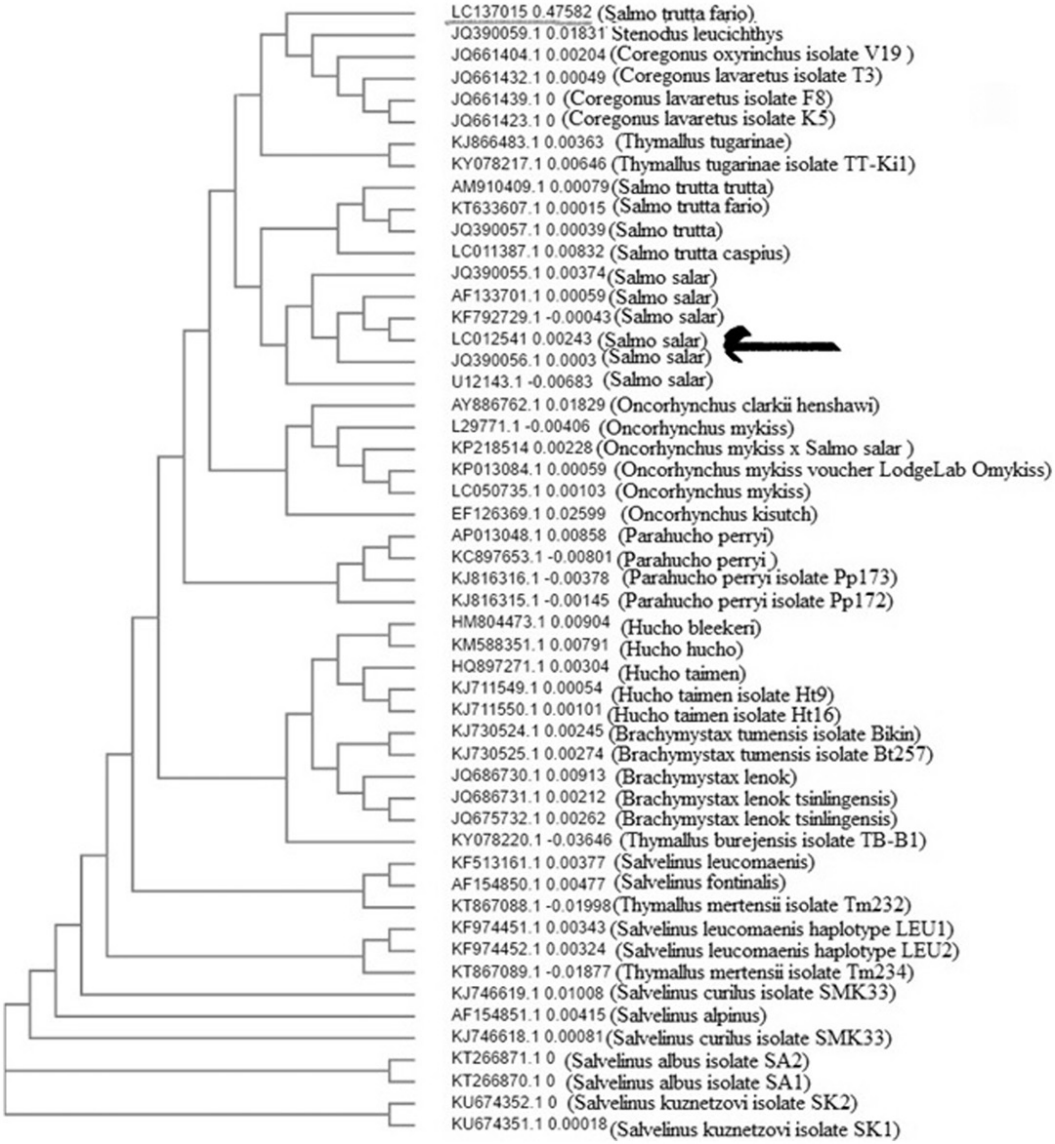
The mitochondrial genomic of *S. salar* (different accession numbers) were compared with MEGA 7.0. The result showed *S. salar* in one clade and another species of salmonids of other clade.
